# Urban Intelligence for Pandemic Response: Viewpoint

**DOI:** 10.2196/18873

**Published:** 2020-04-14

**Authors:** Yuan Lai, Wesley Yeung, Leo Anthony Celi

**Affiliations:** 1 Department of Urban Studies and Planning Massachusetts Institute of Technology Cambridge, MA United States; 2 Laboratory for Computational Physiology Massachusetts Institute of Technology Cambridge, MA United States; 3 National University Hospital Singapore Singapore; 4 Harvard Medical School Boston, MA United States

**Keywords:** urban informatics, urban science, data science, health emergency, medical informatics, COVID-19, pandemic, outbreak, public health, infectious disease

## Abstract

Previous epidemic management research proves the importance of city-level information, but also highlights limited expertise in urban data applications during a pandemic outbreak. In this paper, we provide an overview of city-level information, in combination with analytical and operational capacity, that define urban intelligence for supporting response to disease outbreaks. We present five components (movement, facilities, people, information, and engagement) that have been previously investigated but remain siloed to successfully orchestrate an integrated pandemic response. Reflecting on the coronavirus disease (COVID-19) outbreak that was first identified in Wuhan, China, we discuss the opportunities, technical challenges, and foreseeable controversies for deploying urban intelligence during a pandemic. Finally, we emphasize the urgency of building urban intelligence through cross-disciplinary research and collaborative practice on a global scale.

## Introduction

Cities have become the “locus of risks” due to increasing natural disasters, health pandemics, political protests, and organized crime [1]. Megacities, which the United Nations defines as more than 10 million citizens, face increasing risks in environmental and population health, despite their economic prosperity and status as hubs for cultural exchange and technological innovation. The United Nations predicts that there will be 43 megacities by 2030, with the majority of them in developing countries. By 2050, the world population will be nearly 10 billion, with an estimated 68% living in urban areas [2]. Due to their population density and connectivity, megacities are particularly vulnerable to infectious diseases as seen in the dengue, Zika, and severe acute respiratory syndrome epidemics. Meanwhile, the rapid development of information and communications technology (ICT), Internet of Things, cloud computing, and smartphone apps has enabled near real time information sharing. The large volume, velocity, and variety of urban data enable a deeper and holistic understanding of urban conditions and real time situations.

The unfolding of the current coronavirus disease (COVID-19) pandemic has drawn significant global attention. The outbreak was first identified in Wuhan, a megacity with more than 11 million people in China [3]. The soaring number of confirmed cases and deaths immediately drew serious attention from the medical community to address the pandemic by employing different approaches. Although there is extensive scientific literature on the environmental, social, economic, and health aspects of urban epidemics, most studies focus on long-term planning and public policy research. Studies have revealed that the city-level endogenous differences, including geography, population characteristics, spatial structure, regional connectivity, and microclimate, are associated with variations in epidemic dynamics (eg, transmission potential and infection patterns) across cities [4]. However, few studies have addressed how urban data and data science methodologies can be applied for pandemic response. As an interdisciplinary field, data science provides tools for better and timely information management and data use. In this article, we highlight the urgency to develop city data expertise and data science practice to better design information collection and integrate predictive analytics to implement real time responses in cities. Reflecting on the ongoing novel coronavirus pandemic, we explore two critical questions: What are currently available data in cities? What are the possible uses of urban data for the epidemic response?

First, we define urban intelligence as a capacity that analyzes city-level information using data science methods and explore its role in a pandemic response. In this context, we present five well-investigated urban research areas that are crucial components of urban intelligence in a disease outbreak. Second, reflecting on the current novel coronavirus outbreak, we summarize the opportunities and challenges in the preparation, containment, and recovery from a pandemic. Third, we discuss arguments and debates around uncertainty, privacy, information security, as well as the trade-off between timeliness and accuracy of data exchange during an outbreak.

## Methods, Search Strategy, and Selection Criteria

Data for this viewpoint were identified by searches of PubMed, Social Sciences Citation Index, Science Citation Index, Scopus, and references from relevant articles using the search terms “urban intelligence,” “urban health,” and “pandemic response”. Reports, news articles, or websites were included only when they related directly to previously published work, or they were the only currently available information source at the moment of manuscript preparation. Only articles in English between 1965 and 2020 were included. One Chinese website was cited since it was the only available and most widely adopted media platform during the COVID-19 outbreak in Wuhan in February 2020.

## Defining Urban Intelligence

### Urban Intelligence Capacity

The concept of a “smart city” is arguably new, but there was considerable research done in urban intelligence during the aftermath of World War II. In 1965, Webber [5] proposed “intelligence centers that bring scientific morality into urban affairs” to address an increasing complexity of cities with interactional consequences among transportation, communication, organization, and social behavior. Sternberg describes intelligence as “complex analytics, modeling, optimization, and visualization in the operational business processes to make better operational decisions” [6]. For cities in particular, Kitchin [7] describes urban intelligence as a capacity “to monitor, manage and regulate city flows and processes, often in real-time, and mobile computing, ..., and uses rich seams of data that can used to better depict model and predict urban processes and simulate the likely outcomes of future urban development”. Day and Schuler [8] provide an extended sociotechnical view on civic intelligence with inclusiveness and engagement as “the capacity that organizations and society use to *make sense* of information and events and craft responses to environmental and other challenges collectively”. In summary, intelligence is a capability to collect urban contextual and situational data as digital representations of the reality (input); perceive information from various sources of data (processing); generate knowledge (output); and direct responses, behaviors, or decisions within a specific environment (action).

**Figure 1:**
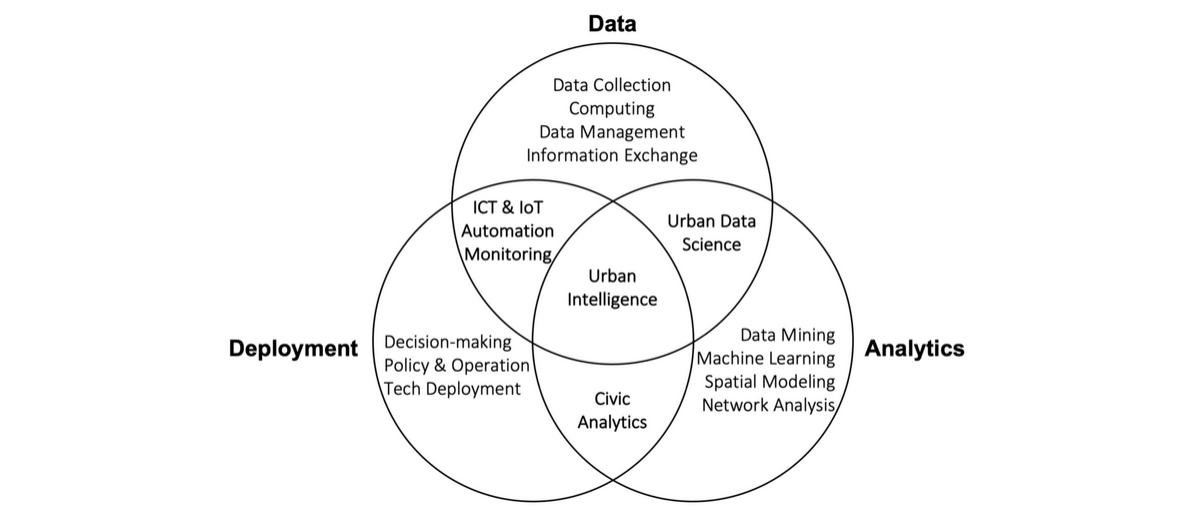
A graphic illustration of urban intelligence core components. ICT: information and communications technology; IoT: Internet of Things.

Figure 1 shows the core components of urban intelligence. We identified three fundamental capacities enabling urban intelligence: city information resources, data science skills, and executive power to operate. Urban intelligence derives from information that requires in-depth knowledge of different sources and types of data in cities, as well as processes for their collection, management, and exchange. The transdisciplinary field is referred to as urban informatics, which encompasses the generation and application of data and related information technology in the context of cities, and lies at the intersection of people, places, and technology [9]. A more specific definition describes it as a study of urban phenomena to address domain-specific urban challenges such as a pandemic response through a data science framework and computational techniques including sensing, data mining, information integration, modeling, analysis, and visualization [10]. Experts in this field collect and analyze data by using a wide range of scientific, engineering, and computational methods, such as sensing (in situ, remote, or mobile sensing), imagery processing, natural language processing, statistical modeling, graph-based network analysis, machine learning, and geographical information system.

The second component of urban intelligence is the analytical capacity using data science. A definition of data science describes it as a discipline of knowledge extraction from data using computer science, statistics, and domain expertise [11]. The distinction of data science from conventional statistics is its capacity for handling a much larger volume of heterogeneous and unstructured data [12]. Most experts in big data computing, machine learning, and artificial intelligence are proficient in computer science and statistics, but there are few who also have domain knowledge. Domain expertise is essential for identifying actionable insights (feasibility for deployment and measurable improvement of actual operation), validating meaningful predictions (including its accuracy, sensitivity, and relevance to decision making), and evaluating potential impact (eg, expected and unexpected social, economic, and political consequences). The last, arguably the most crucial component for urban intelligence in the context of a pandemic, is the emergency operational and executive power for critical event preparedness and response.

### Urban Intelligence During a Pandemic

We present five components of urban intelligence (movement, facilities, people, information, engagement) that are well-researched but remain too siloed to successfully orchestrate an integrated pandemic response. Table 1 summarizes specific data sources, analytical tasks, and actions to take during a pandemic.

**Table 1:** A summary of five critical urban intelligence components for a pandemic response.

Components	Data sources	Analytical tasks	Actions and operations
Movement	Air flights, ground transportation, GPS tracing, cellphone pings	Identify mobility hot spots and develop network algorithms to analyze spatial patterns and flows	Transportation control, checkpoints, identify quarantine zones, contact tracing
Facilities	Facility catalog, resource inventory, infrastructure performance	Model capacity and optimize medical staffing and resource triaging	Logistical distribution and human resources, capital planning
People	Population census, community survey	Quantify local population characteristics and neighborhood health baseline measures	Provide additional services to vulnerable population groups and communities
Information	User agreement and protocols for data exchange	Develop an information exchange and coordination pipeline during a pandemic	Integrate and manage data across various resources and agencies
Engagement	Digital platforms, news and social media, open data portal	Identify high influencers on social media and less active sectors or regions that require proactive outreach	Broadcast news and crowdsource local information

### Movement

Quantification of spatial connectivity and mapping real time human mobility at the intraurban scale provide actionable insights for a pandemic response. The impact of geography on epidemic dynamics and pandemic transmission hubs at the regional scale have been reported [4,13], but there are limited investigations in inter- or intraurban connectivity and human mobility. The human movement between cities needs better data sources and quantification methods. Conventional data such as population census or community survey reveal regional connectivity and spatial structure of the human movement. Real time or near real time human mobility data during a pandemic is valuable since regional population movement may unmask abnormal behavior during a critical event. In a study on the COVID-19 outbreak in Wuhan, the research team measured intercity connectivity by using three data sources, including global flight bookings from the Official Aviation Guide, the prefecture-level daily passenger volume (by transportation modes) based on location-based services provided by Tencent (one of the largest information technology companies and the operator of WeChat), and the historical estimation of Spring Festival travelers reported by the municipal transportation department [14]. Such information may guide national pandemic forecasting and regional interventions (eg, reschedule the flights and high-speed railway operations); however, it does not provide intracity human mobility for modeling complex spatial-temporal patterns. Relevant data includes human mobility trajectory mapping based on cell phone pings [15], real time local population estimation using public Wi-Fi probe [16], intracity mobility pattern detection using GPS loggers or General Transit Feed Specification data provided by buses, taxis, and bike-share operators [17,18], and spatial analysis of human-scale economic and social activities using geotagged social media feeds (eg, Twitter, Instagram, Foursquare, Yelp) [19-21]. During a pandemic, all these data provide more spatially specific indications for containment strategy and inform contact tracing.

### Facilities

City agencies collect and manage information on large data inventory of critical facilities and resources. A study from Johns Hopkins University evaluated hospital surge capacity for maintaining basic operation (eg, sanitation, food, communication, security) and standards of care during four catastrophic scenarios (pandemic influenza, radiation, explosive, and nerve gas attack). In a pandemic influenza scenario particularly, the top five critical facilities and resources are: isolation room or cohorting, respiratory therapist, face masks, antiviral agents for influenza, and dialysis [22]. Besides public health systems, other facilities owned and operated by cities play supportive roles during a pandemic. The New York City Department of City Planning manages the City Planning Facilities Database with more than 35,000 city, state, or federal-owned facilities and program sites, including public schools, daycare service providers, public libraries, parks, sports stadiums, and recreational centers [23]. The initial purpose of this data inventory is for public budget allocation, neighborhood funding evaluation, and capital planning, but data reporting the location and capacity of facilities provide valuable information for disaster planning and response. Private mapping or navigation application program interfaces, including Google Maps, TomTom, and Foursquare, provide near real time information on geolocation and operations (business hours and peak hours). Studies on sensor data applications and computing in the urban environment have identified points of interest (POI) as one of the critical measures for estimating local human activity and related exposure risk for disaster management [24,25]. Besides public-owned facilities, POI in cities, such as local clinics, drug stores, convenience stores, and grocery shops, become critical nodes and suppliers to ensure uninfected people’s well-being during the outbreak.

### People

Neighborhood demographics including socioeconomic profiles provide essential baseline measures for identifying underlying infection risks based on population characteristics. One example is the New York City Community Health Profiles, a census on 59 community districts of population health, reporting more than 50 metrics on neighborhood environmental and population health along with social and behavioral indicators (eg, education, income, smoking, alcohol consumption) [26]. The initial purpose of the data collection is to quantify neighborhood health and quality-of-life metrics, but population data by specific age groups (eg, infants, children, or older adults), health condition, and socioeconomic status identify vulnerable communities. Recent research and practice have proven that urban data sources with high spatial resolution can support better operations that target specific population groups such as children, older adults, and the homeless population [27]. Beyond estimating the vulnerable population at risk for infections, ICT can provide educational and other care services for older adults or “left-behind” children at the community scale [28-30]. Under a data governance that protects information security and respects personal privacy, these additional data can inform city agencies, social institutions, and community-based organizations to provide local and targeted services during a pandemic.

### Information

During the Obama administration, the Open Government Partnership focused on the role of integrated data and urged city agencies to generate cross-cutting initiatives and data exchange protocols [31]. As a result, interorganizational institutions for better information integration, such as the New York City Center for Innovation through Data Intelligence, were created [32]. A citywide interagencies data exchange protocol is crucial to inform public and private sectors on who collects what data and for what purposes. Better data exchange will reduce response time and information discrepancy to mobilize resources and coordinate multiagency operations (eg, an outbreak in a public school may require both the health and education departments). Data exchange protocol also optimizes delegation of duties at different levels of urgency during a pandemic. During the 2009 H1N1 pandemic, NYC 311, a citywide agency managing nonemergency service requests, received and triaged approximately 54,000 phone calls regarding possible influenza [33]. In a pandemic situation, interagency coordination does not only improve information exchange but also appropriately triages health care service response for a more efficient operation.

### Engagement

Active and productive engagement between city agencies and the general public plays a vital role during a pandemic outbreak. Government-citizen communication and social media analytics can raise people’s awareness, monitor public sentiment, and identify false alarms or fake news. During the 2009 H1N1 pandemic in Mexico City, Telmex, a major telecommunication operator in Mexico, managed more than 5 million phone calls, 140 million text messages, and 18 million email messages containing official communications from the Ministry of Health [33]. Besides the traditional telecommunication services, social media platforms play a critical role in broadcasting news and promoting preventive actions to the general public. Crowdsourcing data collection provides a unique value for infectious disease surveillance [34]. Crowdsourcing provides alternative information when no other data are available, improves the spatial-temporal resolution of disease analysis with geotagged high-frequency data, and increases public health awareness through the participatory process. During the COVID-19 outbreak, Ding Xiang Yuan (DXY), a leading digital health platform in China, provided a stage for broadcasting real time information and public engagement [35]. The platform had three components: real time mapping of confirmed cases and deaths, dispelling rumors and fake news, and public education on prevention. By combining crowd-sourced data on the DXY platform with data from news sources and national health agency websites, researchers were able to gather information that would be otherwise difficult to obtain from aggregate data released by health authorities, such as the delay between symptom onset and detection by the health care system, reporting delays, and travel histories [36]. Engagement also mitigates the massive societal disruption during the outbreak. Multiple service companies have emerged that offer platforms to support virtual offices and online education. Such platform-based services are critical for reducing unnecessary travel demands and physiological stress during the quarantine.

### Challenges

Information transparency remains a major issue, but there is a parallel issue of the disconnect between information and data. In recent years, Chinese public information platforms and mobile phone apps have generated massive amounts of data. During the COVID-19 outbreak, the majority of information was released in the format of news, texts, infographics, tables, or map images for public disclosure purposes only, leading to limited data for computation. Chinese public officials regularly shared information of high-speed rail departures and aircraft flights with suspected cases, but as infographics through social media (WeChat or Weibo). Even when information was released, no publicly available data was provided due to a lack of data standards or guidelines. Although crowdsourcing has become an accepted approach for collecting data on a large scale, the quality and consistency of data collection remain a challenge due to its participatory nature. Social media provides near real time information on newly confirmed cases, but it is necessary to consider the trade-offs between timeliness and accuracy. Machine learning and deep learning that train on retrospective data are promising for artificial intelligence-assisted diagnoses and other population health tasks, but they currently have limited real time practical value. As of early February 2020, almost 2 months since the COVID-19 outbreak, the most comprehensive research resource only had 1334 patient-level records [37].

Even for data-savvy cities such as New York City or Singapore, heterogeneous data sources produce messy and typically biased data from a lack of representativeness. These limitations create so-called “signal problems” that skew the understanding of reality [38]. As the former Director of Analytics of New York City described, a fire hose of information is of no use unless it points to a fire [39]. During a pandemic, information initiatives require tremendous cross-disciplinary knowledge, resources, network, and a political willingness to connect and link data with the right people with domain expertise for the right problems.

Insights do not guarantee actions. Data scientists are prone to be “paralyzed by analysis,” while ground operations in a real world urban environment must be responsive, proactive, and agile to act with incomplete data and missing information. Moral dilemmas around optimization criteria, the liability associated with uncertainties, and concerns around unexpected public reactions, engender social, technical, and political challenges for transforming insights into actions. Privacy threats, cybersecurity vulnerability, ethical controversies, and unanticipated societal impact further create risks for scaling urban intelligence.

## Conclusion

The impact of a pandemic is far beyond public health and medical care. It brings large scale economic risks and social instability, especially for densely populated megacities. Expertise in the generation, collection, analytics, and application of urban data can bring tremendous value to support better response and prevention during a pandemic. Even when a situation gets effectively controlled, urban intelligence can provide continuous risk assessment and support economic recovery. As we continue to face an unprecedented pandemic, we need to identify and implement best practices in urban intelligence to define the critical role of cities in the global public health crisis.

## Abbreviations

COVID-19: coronavirus disease
DXY: Ding Xiang Yuan
ICT: information and communications technology
POI: point of interest
